# A Rare Case of Malignant Plasmacytoma at a Previous Injection Site in a Cat

**DOI:** 10.3390/vetsci13040384

**Published:** 2026-04-16

**Authors:** Hasuk Nam, Yeon Chae, Yoonhoi Koo, Hakhyun Kim, Byeong-Teck Kang, Taesik Yun

**Affiliations:** 1 Laboratory of Veterinary Internal Medicine, College of Veterinary Medicine, Chungbuk National University, Cheongju 28644, Republic of Korea; threetap@naver.com (H.N.); bluesfiddle@naver.com (Y.C.); kimh@chungbuk.ac.kr (H.K.); kangbt@chungbuk.ac.kr (B.-T.K.); 2 College of Veterinary Medicine, Kyungpook National University, Daegu 41566, Republic of Korea; yoonhoi@knu.ac.kr

**Keywords:** feline, injection site, plasmacytoma, vaccination, immunohistochemistry, MUM-1

## Abstract

A 6-year-old castrated male Bengal cat developed a subcutaneous mass at a previous vaccination site, which was initially diagnosed as cutaneous T-cell lymphoma following surgical excision at a local hospital. The cat was referred for metastatic evaluation and further treatment. Computed tomography revealed a residual tumor at the excision site, and an additional thoracic subcutaneous mass was identified. Both lesions were surgically excised and submitted for histopathologic and immunohistochemical examination. Despite the initial diagnosis of lymphoma, immunohistochemistry demonstrated that the neoplastic cells were negative for multiple lineage-specific markers, including CD3, Pax5, CD20, and cytokeratin, but showed strong MUM-1 positivity in mitotically active cells. These findings supported a diagnosis of plasmacytoma. This case emphasizes a diagnostic challenge in distinguishing round cell tumors in cats and the critical role of immunohistochemistry in achieving an accurate diagnosis. The tumor also showed aggressive clinical behavior, including suspected distant metastasis, and may have arisen in association with chronic inflammation at a prior injection site.

## Simple Summary

This report describes a rare and serious tumor, a plasmacytoma, found in a 6-year-old Bengal cat. The tumor developed at the site where the cat had previously been vaccinated. Although most skin tumors of this type in cats are harmless and can be easily removed, this tumor behaved differently. It grew rapidly, showed signs of spreading to other parts of the body, and was challenging to treat. Veterinarians used special tests to determine the exact type of tumor. Their thorough investigation revealed that these tumors can sometimes act much more aggressively than expected, especially when they occur at injection sites. Learning from this case can help veterinarians and pet owners recognize unusual signs early and select the best treatment options for cats with skin lumps or bumps.

## 1. Introduction

Plasmacytoma (PCT) is a type of round cell tumor (RCT) that originates from terminally differentiated B lymphocytes [1]. Myeloma-related disorders, characterized by clonal neoplastic proliferation of plasma cells, include multiple myeloma, solitary osseous PCT, and extramedullary plasmacytoma (EMP). In small animals, EMP is generally considered a benign neoplasm that is usually cured after surgical excision. Although the incidence of feline PCT has not been clearly established, it is considered extremely rare, as myeloma-related disorders account for only 0.003% to 0.1% of all feline malignancies [2]. However, the malignant potential of EMP in cats has not been well characterized.

Several studies have shown that injections are associated with an increased incidence of tumors in cats [3,4]. Although feline injection-site sarcomas (FISS) are the most frequently encountered form of injection-associated tumor in cats, a few cases of cutaneous lymphomas at injection sites (CLIS) have also been reported. These studies have suggested that chronic inflammation induced by vaccination or injection procedures can contribute to tumor development, as reported in FISS and CLIS [3,4,5].

RCTs are characterized by populations of cells with round nuclei and distinct cytoplasmic borders [1]. In many cases, due to the similar morphology of RCTs, cytologic and histopathologic evaluation alone may not be sufficient to obtain a definitive diagnosis, particularly when the neoplastic cells are poorly differentiated [6]. Therefore, immunohistochemistry is needed for the accurate diagnosis of PCT. Markers of terminal B-cell differentiation, such as MUM-1, are essential for confirming plasma cell origin [7,8]. Since confirmation of plasmacytic differentiation often requires a panel of immunohistochemical markers and stepwise exclusion of other RCT lineages, establishing a definitive diagnosis may be challenging in clinical practice [9].

In this case report, we present a rare case of malignant PCT that developed at a prior vaccination site in a cat. This case suggests the potential for aggressive clinical behavior in plasmacytomas, which are generally considered benign, and a possible relevance of chronic inflammation at an injection site in the development of this tumor. In addition, this case demonstrated the diagnostic value of immunohistochemistry in the evaluation of morphologically ambiguous RCTs.

## 2. Case Presentation

A 6-year-old castrated male Bengal cat presented for metastasis screening of an interscapular cutaneous mass initially identified at a local animal hospital. The cat had a history of vaccination at the same site approximately two years before presentation. The mass was surgically excised at a local hospital and submitted to an external laboratory for histological examination, where it was diagnosed as cutaneous T-cell lymphoma. The original histopathology slides and immunohistochemical results were not available for review, precluding assessment of surgical margins from the initial excision.

**Figure 1:**
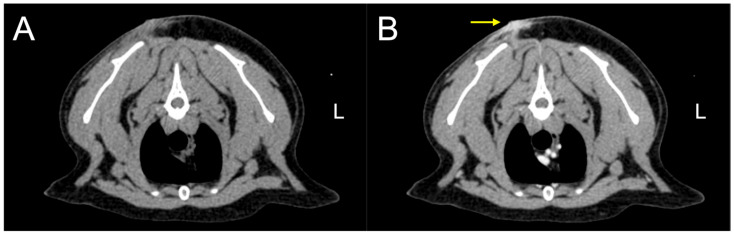
Computed tomography transverse images of the thoracic cavity before (**A**) and after (**B**) contrast in a cat with plasmacytoma. The computed tomography images show a contrast-enhancing lesion within the cutaneous and subcutaneous layers (arrow).

At presentation, the cat was alert, and the complete blood count and serum chemistry were unremarkable. The computed tomography revealed a subcutaneous mass in the interscapular region without radiologic evidence of visceral or mediastinal involvement (Figure 1). Revision surgery was planned a month later. During the preoperative physical examination, a thoracic mass was newly detected. Both dorsal and thoracic masses were surgically excised and submitted for polymerase chain reaction for antigen receptor rearrangement (PARR), histopathologic, and immunohistochemical examinations.

While awaiting the histopathology and PARR results, adjuvant chemotherapy was initiated based on the previous diagnosis of cutaneous T-cell lymphoma at a local hospital. Lomustine (40 mg/m^2^; CeeNU^®^ Bristol-Myers Squibb Co., Ltd., Princeton, NJ, USA) and prednisolone (2 mg/kg BW, once daily, PO; Solondo^®^, Yuhan, Seoul, Republic of Korea) were prescribed as chemotherapy agents.

**Figure 2:**
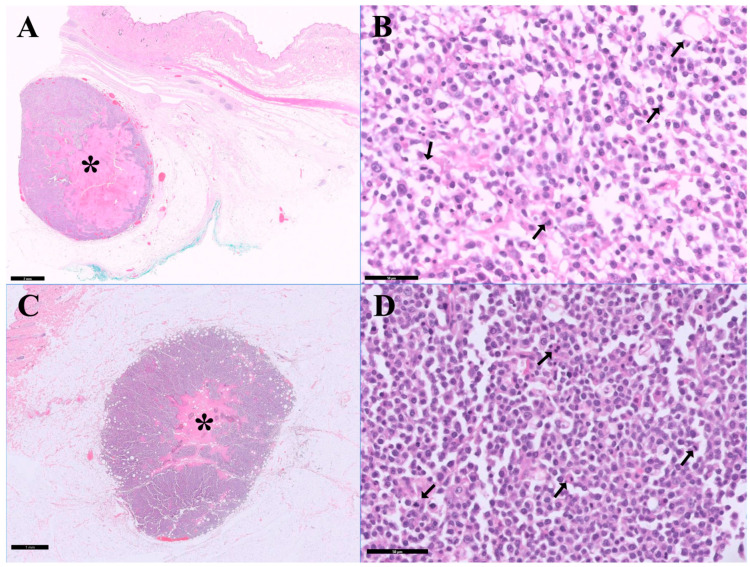
Histopathological examination of the surgically resected dorsal and thoracic lesions in a cat with plasmacytoma: (**A**) Dorsal mass, low-magnification view showing a solitary, densely cellular mass (asterisk) arising in the subcutaneous tissue with expansile growth and infiltration into adjacent adipose tissue, supported by fibrous stroma, with focal necrosis and inflammatory infiltrates (scale bar = 2 mm). (**B**) Dorsal mass, high-magnification view demonstrating a sheet of round neoplastic cells with scant basophilic cytoplasm, centrally located round to oval nuclei, coarse chromatin, and occasional prominent nucleoli; frequent mitotic figures (arrows) were observed (scale bar = 50 µm). (**C**) Thoracic mass, low-magnification view showing similar densely cellular proliferation (asterisk) within the subcutaneous tissue (scale bar = 1 mm). (**D**) Thoracic mass, high-magnification view demonstrating neoplastic round cells with similar cytologic features and frequent mitotic figures (arrows) (scale bar = 50 µm). Hematoxylin and eosin staining.

The PARR and hematoxylin and eosin staining results were reported before immunohistochemistry (Figure 2). The PARR results revealed clonal rearrangements in the T-cell receptor gamma gene (91 and 102 bp) and in the immunoglobulin heavy-chain gene (251 and 171 bp), with a moderate polyclonal background. Histopathologic examination of the dorsal and thoracic skin masses demonstrated focal proliferations of neoplastic round cells, most consistent with nonepitheliotropic large cell lymphoma. However, based on PARR and histopathologic findings, the lineage of the neoplastic cells could not be definitively established, and definitive classification required immunohistochemical evaluation.

After the initiation of chemotherapy, the cat developed right-sided facial nerve paralysis, characterized by the absence of menace response and palpebral reflex on the affected side, while corneal and gag reflexes remained intact. Based on these findings, magnetic resonance imaging was recommended to differentiate a primary neurologic condition from possible metastatic involvement of the central nervous system. During pre-anesthetic evaluation, a pulmonary nodule was identified on thoracic radiographs. With suspected metastatic involvement of both the lung and central nervous system, the owner refused any further diagnostic tests, including magnetic resonance imaging.

**Figure 3:**
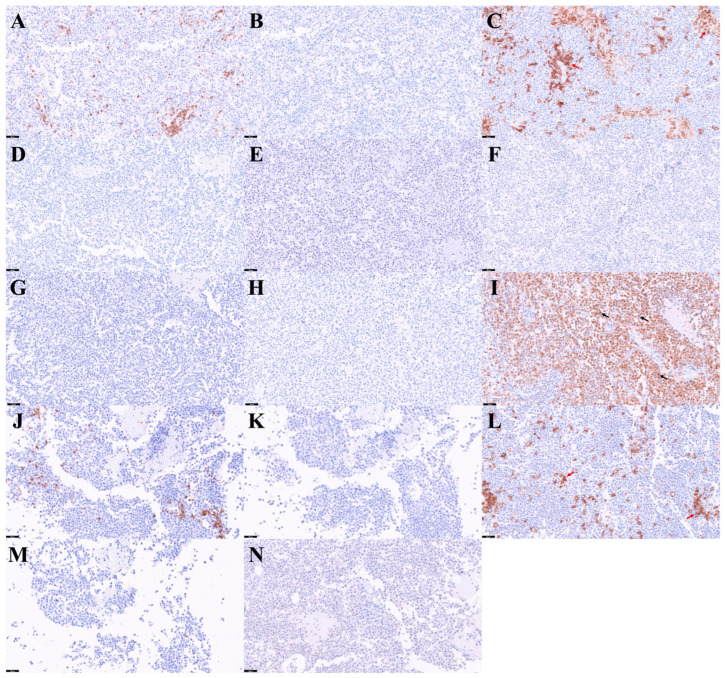
Immunohistochemical examination of the surgically resected dorsal cutaneous lesion in a cat with plasmacytoma: (**A**–**I**) Representative sections from the dorsal mass show negative immunoreactivity for CD3 (**A**), Pax5 (**B**), CD18 in neoplastic cells with scattered positive cells in the periphery (red arrows) (**C**), CD20 (**D**), CK20 (**E**), synaptophysin (**F**), cytokeratin AE1/AE3 (**G**), and SATB2 (**H**), while neoplastic cells show positive immunoreactivity for MUM-1 (arrows) (**I**). (**J**–**N**) Representative sections from the thoracic mass show negative immunoreactivity for CD3 (**J**), Pax5 (**K**), CD18 in neoplastic cells with scattered positive cells in the periphery (red arrows) (**L**), CD20 (**M**), and synaptophysin (**N**), consistent with the immunophenotype observed in the dorsal mass. Scale bars: (**A**–**N**) 50 µm.

The cat died approximately three months after revision surgery. The full results of immunohistochemical analysis were reported after death. The immunohistochemical panels are presented in the order in which they were applied (Figure 3). Immunohistochemical evaluation demonstrated that the neoplastic cells were negative for multiple lineage-specific markers, including CD3, Pax5, CD18, CD20, CK20, synaptophysin, cytokeratin AE1/AE3, and SATB2, thereby excluding T-cell, B-cell, epithelial, and neuroendocrine differentiation (Figure 3A–H). In contrast, most neoplastic cells exhibited strong nuclear immunoreactivity for MUM-1 (Figure 3I), supporting a diagnosis of plasmacytoma. CD18 immunoreactivity was observed only in scattered peripheral cells and not in mitotically active cells (Figure 3C).

The thoracic mass demonstrated histopathologic features similar to those of the dorsal lesion. Immunohistochemical evaluation of the thoracic mass, including CD3, Pax5, CD18, CD20, and synaptophysin (Figure 3J–N), showed a similar immunophenotypic profile, with negative staining in neoplastic cells and CD18 positivity in scattered peripheral cells consistent with the findings in the dorsal mass. Based on these concordant findings, additional immunohistochemical markers were not further evaluated in the thoracic lesion.

## 3. Discussion

This case is noteworthy for three main reasons. First, the tumor exhibited aggressive clinical and histopathologic characteristics, whereas EMPs in small animals are generally considered benign and often curable by surgical excision. Second, the tumor developed at a previous injection site and represents a non-sarcoma neoplasm in this setting, where tumors are most commonly represented by FISS. This finding expands the spectrum of tumors reported at feline injection sites. Third, this case demonstrates the diagnostic challenges of poorly differentiated RCTs and emphasizes the value of immunohistochemistry in establishing a definitive diagnosis.

Myeloma-related disorders are characterized by clonal neoplastic proliferation of plasma cells as previously described [1]. Multiple myeloma is considered the archetypal disease within the spectrum of myeloma-related disorders and is typically associated with systemic involvement, including bone marrow infiltration and related clinicopathologic abnormalities [10]. In contrast, EMPs have generally been reported as localized lesions without evidence of systemic disease [1,11]. In the present case, the tumor exhibited a high mitotic index compared to previously reported feline cutaneous EMPs [12]. In addition, a second lesion in the thoracic region showing similar histopathologic and immunohistochemical features was identified, suggesting that the two lesions may represent the same neoplastic process, although definitive confirmation was not obtained.

Tumors arising at previous injection sites in cats are most commonly represented by FISS. However, rare non-sarcoma tumors, including lymphomas arising at injection sites (CLIS), have also been described [4,13]. In this context, the present case represents a PCT arising at a previous injection site and therefore expands the spectrum of tumors reported at feline injection sites to include PCTs. Chronic inflammation has been proposed as a contributing factor in the development of tumors at injection sites [4,13,14]. The interval between injection and tumor development in this case was approximately 2 years. This latency period is consistent with ranges reported for FISS (4 months to 3 years) and CLIS (15 days to 9 years) [4,13].

In both FISS and CLIS, chronic inflammation induced by injection may have contributed to tumorigenesis at injection sites [4,14]. A similar mechanism may be considered in the present case. CD18 immunolabeling was observed predominantly at the tumor periphery rather than in mitotically active cells. CD18 is a marker of macrophage and mononuclear phagocytic lineage, and its expression is consistent with peritumoral macrophage infiltration and supports the presence of surrounding chronic inflammation [15]. These findings raise the possibility that the tumor shares pathogenetic features with FISS and CLIS. The pathogenesis of injection-site-associated neoplasia has been suggested to involve chronic antigenic stimulation and subsequent alterations of the local inflammatory microenvironment. Persistent inflammation induced by vaccines or adjuvants may contribute to chromosomal instability and promote neoplastic transformation [11]. In addition, prolonged inflammatory responses have been associated with increased production of cytokines such as interleukin-6 and interleukin-10, which may enable transforming B cells to escape from host immune surveillance [4].

Diagnosing the PCT was challenging. The PARR results demonstrated dual clonal rearrangements involving both T-cell receptor and immunoglobulin heavy-chain genes. These findings should be interpreted with caution, as they may occur as artifacts in the presence of a polyclonal inflammatory background [16]. Inflammatory lymphocyte infiltration may further contribute to this pattern, and the PARR findings were therefore insufficient to determine tumor lineage [16]. These findings support a recognized limitation of PARR and emphasize the need for correlation with histopathologic and immunohistochemical results.

In this case, sequential immunohistochemical analyses were ultimately required, and the entire diagnostic process extended over approximately three months following the revision surgery, despite evaluation by a board-certified veterinary pathologist. Unfortunately, the cat died before the diagnosis could be definitively confirmed. This delay was likely influenced by the initial external diagnosis of T-cell lymphoma, which could not be independently reviewed. In addition, the histopathologic findings were most consistent with nonepitheliotropic large cell lymphoma, characterized by a densely cellular, sheet-like proliferation of round cells with a background of small mature lymphocytes, and immunohistochemical panels were therefore initially directed toward confirming this diagnosis, with additional markers applied in a stepwise manner [9].

In this case, positive MUM-1 immunoreactivity was critical for establishing plasmacytic differentiation after other major RCT lineages had been excluded [7,8]. This finding suggests that immunohistochemistry should be considered as a first-line diagnostic tool in the evaluation of poorly differentiated RCTs, as cytologic and histopathologic evaluation alone may not be sufficient due to overlapping morphology. In particular, early application of plasma cell markers such as MUM-1 may facilitate more rapid and accurate diagnosis, rather than relying solely on stepwise testing approaches.

There are certain limitations in this case. First, although the definitive diagnosis was established through immunohistochemistry, the cat died before any treatment could be initiated based on this result. Second, although detailed information regarding the type of vaccine and the exact number of administrations was not available, the owner reported that repeated inoculations had been administered at the same site. The anatomical location and clinical history therefore support a possible association with injection-site PCT development. Third, neurological signs were considered suspicious for central nervous system involvement, and a pulmonary nodule was identified. However, it could not be determined whether these findings were related to the present tumor, including the possibility of metastatic disease. Fourth, chemotherapy was initiated based on a previous diagnosis of cutaneous T-cell lymphoma at a local hospital. Since limited information was available, initiation of chemotherapy based on the presumptive diagnosis of T-cell lymphoma was considered a clinically reasonable approach at the time. However, as plasmacytomas are typically managed with alternative therapeutic approaches, including melphalan-based protocols, earlier diagnostic confirmation may have facilitated more appropriate treatment selection [17].

Despite these limitations, this case provides several clinical implications. First, PCTs arising at previous vaccination sites in cats may exhibit more aggressive biological behavior than is typically described for extramedullary PCTs. Second, the development of this tumor at an injection site suggests a possible contribution of chronic inflammation, similar to the pathogenesis proposed for FISS and CLIS. Finally, PCT should be considered among the differential diagnoses for feline injection-site-associated tumors, especially round cell neoplasms. Recognition of this tumor type is essential for accurate diagnosis and appropriate therapeutic planning, including consideration of alternative therapeutic approaches such as melphalan-based protocols.

## Data Availability

The original contributions presented in this study are included in the article. Further inquiries can be directed to the corresponding author.

## Abbreviations

The following abbreviations are used in this manuscript:

PCT	Plasmacytoma
RCT	Round cell tumor
EMP	Extramedullary plasmacytoma
FISS	Feline injection-site sarcoma
CLIS	Cutaneous lymphomas at injection sites
PARR	Polymerase chain reaction for antigen receptor rearrangement
